# Model for implementation of a modern journal club in medical physics residency programs

**DOI:** 10.1002/acm2.13250

**Published:** 2021-05-13

**Authors:** Ashley J. Cetnar

**Affiliations:** ^1^ Department of Radiation Oncology The Ohio State University Columbus OH USA

**Keywords:** discourse, education, journal club, medical physics, medicine

## Abstract

Journal clubs are a common educational experience for medical physics residents as a forum to discuss current research within the field. While journal clubs are valued by educational programs and accrediting bodies, there are a wide variety of ways in which these sessions are conducted. Unfortunately, there are currently few studies that have assessed the effectiveness of this educational method. This review defines journal club in the context of a medical physics residency and provides historical background for the meetings. Reasons why journal clubs are valued are presented, and several methods are described for conducting journal clubs. The format of journal clubs and scaffolding methods for guiding residents in gaining independence in critical reading skills are discussed. While the traditional journal club is a meeting, an alternative online virtual journal club is also described. Finally, a model of how a journal club can be applied in a medical physics residency is presented.

## 1 INTRODUCTION

“We now accept the fact that learning is a lifelong process of keeping abreast of change. And the most pressing task is to teach people how to learn.”^1^

After the completion of graduate school, many medical physics students transition into a residency program where they spend time gaining in depth knowledge of the clinical aspects of their specialty. Since the shift is from didactic training to an apprenticeship model, the medium for education shifts as well. One of the components found in almost every medical physics residency is a journal club. While journal clubs are valued by educational programs and accrediting bodies, there is a wide variety of ways in which these sessions are conducted. There are currently few studies that have assessed the effectiveness of this educational method. The goal of this paper is to present reasons why journal clubs are valued, what methods are used for conducting journal clubs, how discourse is used in these educational settings, and a model for implementing a journal club program within a residency.

### 1.1 Historical background

While the earliest beginnings of the notion of journal club are somewhat unclear, the first recorded meetings were started by Sir James Page in London between 1835 and 1854 and Sir William Osler at McGill University in 1875.^2^ The clubs were originally established to provide a means for the medical community to read about the newest publications since texts were very expensive and the literature could not be easily accessed. These meetings would take place in venues outside of the clinic, including meeting above baker's shops, in restaurants, bars, and faculty members’ homes. This provided an atmosphere for the group to read, evaluate, and discuss the advancement of medicine.

### 1.2 Modern definition of journal club

Now, journal clubs commonly take place within the clinic or hospital during the workday as open meetings for residents, faculty, and staff. Modern journal clubs are defined as educational forums for discussing new scientific research and critically evaluating its applications for improving patient care.^3^, ^4^, ^5^ Skills desired as an outcome of these meetings include increasing residents' critical reading skills and gaining knowledge within the medical specialty.^6^ With a recent focus on evidence based medicine, there is an interest in discussing articles that focus on research that provides data that will impact clinical practice.^7^ By emphasizing realistic problems, residents are often encouraged to lead small group discussions, incorporate interactive learning, or provide a forum for academic discourse within the journal club setting compared to those which primarily consist of lecture based seminars.^8^, ^9^

### 1.3 Models of journal club

While journal clubs have been established in the medical community, some have extended the model into other areas of education including teaching science to undergraduates. Similarities between the two settings include the focus on increasing scientific literacy, providing a venue to effectively communicate ideas, applying research from the literature, and developing critical thinking. However, the main differences are found in the expectations for the students based on their education and the format of how the journal clubs are conducted.

A traditional approach for beginning a journal club involves asking a student to read an article. However, students often approach the task unprepared to analyze the text since expecting a novice to comprehend a technical article can be frustrating to an individual has not been trained to do so. The consequence of this model is that it has the potential to decrease one's enthusiasm for scientific research.^10^ General techniques for approaching scientific research articles involve strategies such as starting with reading the abstract first, followed by the introduction, and results so that the information is more manageable.^11^ Strategies for building confidence can also include a guideline of questions in the form of a worksheet to help students develop these critical reading skills. The instructor can also select a provocative topic that would be of interest and provide information for the students to focus on preselected articles to guide the inquire process in attempt to answer the question at hand.^10^

Roberts^12^ shares a scaffolding framework to help educate students in building the skills needed to present and discuss scientific research. The teacher selected a topic where the students in the class had common ground, so there was the ability for all of the students to contribute. Parts of the journal club include learning how to use search engines, distinguishing quality articles, and presenting scientific work.

Since there is typically little guidance on how students to evaluate literature, Robertson^13^ presents a systematic method for how to interpret research at the undergraduate level. The model is based on four progressive steps beginning with the student developing a personal outline based on the scientific method. This guide is then applied to a sample article that is discussed by the class. Once the student's outline has been tested and revised, it is applied to an article of his choice and the analysis is presented to the class. This method helps students develop both effective communication and scientific literacy one step at a time.

The literature from the undergraduate perspective provides some insightful methods of helping students prepare for the future by scaffolding their skills in scientific literacy and their capacity to integrate new ideas into application. While there are several varying definitions and applications of the term “journal club,” the remainder of this paper will focus on the definition in the context of medical physics residency.

### 1.4 Format of Journal Club

It is recommended by AAPM that clinical medical physics residency training programs provide opportunities for structured conferences and opportunities for participation in scholarly activities including the recommendation inclusion of residents to presentation in journal clubs.^14^ The primary accrediting body for medical residencies, Accreditation Council for Graduate Medical Education (ACGME), requires documentation for residents to display skills of evidence based medicine. Journal club prevails across institutions as the structured learning activity for displaying these skills. However, it is recorded that there is much variation in how these educational experiences are organized from institution to institution.

#### 1.4.1 Goals of journal club

As many as 42% of journal clubs do not have well‐defined goals for their program,^15^ thus it is important to establish clearly defined goals. While a primary goal of original journal clubs was to provide access to the latest innovations and methods for practice, the scope has more recently been extended as not only a means of gaining knowledge but also a way to develop necessary skills that will be helpful professionally, encourage application of research in clinical practice, and provide a means of formal evaluation during the residency.

The initial goal of learning the latest research, practices, and technology is still a primary goal of the journal club. However, it is also an avenue for learning valuable professional skills. The first of these skills is being able to use computer and internet literacy to search for and retrieve a quality scientific article. Second is the ability to read and think critically to analyze scientific literature. Being able to critique research affords residents the chance to develop their reading skills, comprehend the content of journal articles, and create a deeper understanding of research design. By understanding the research presented, the residents are also able to gain specific skills like biostatistical knowledge for evaluating how the data were interpreted within the article.^4^, ^16^ Finally, by having to formally articulate and summarize current research, the resident is able to develop presentation skills.

Reading the article is not enough for practicing evidence‐based medicine. It also requires the residents to find ways to utilize the research in clinical application.^3^ This translation of the data to improving the health of patients should be a primary goal of journal clubs. By having a discussion with other colleagues in this forum, there should be ideas, if not consensus, in ways that the current research can be used to make decisions for improvements within the clinic. This collaborative discussion and approach to critically analyzing literature can be a model for lifelong learning and maintenance of certification.^17^

Residents are evaluated in many ways throughout their daily interactions with the clinical team. However, one of the formal methods for progress and competency is through their presentations in journal club.^3^ Residents that were given an opportunity to practice reading scientific articles and a venue for communicating this understanding with others in the department were found to have increased self‐confidence in solving new and unfamiliar problems which is a critical skill in medicine.^6^ Residents also reported to having better reading habits, being more critical of journal content, and being more skeptical of an author's conclusion as a result of participating in journal clubs.^15^

#### 1.4.2 Logistics

Unlike the original journal clubs from a century ago, most journal clubs are conducted at the work place. They are commonly held before the clinic starts or during lunch or dinner to promote attendance. However, there are still some groups that meet regularly outside of the clinic. It has been noted that the longevity of journal clubs has been associated with several factors including presenting original articles, meeting regularly, and scheduling around a meal.

It has been shown that having regularly scheduled meetings emphasizes the importance of journal club to the department.^4^ While the most common frequency for journal club meetings is monthly, some institutions meet as often as every week. The minimum recommended number is four journal club meetings per year. However, the frequency ultimately depends on the goals of the meetings. It is critical to define why the defined approach to conducting the journal club is valued and why time is being spent in this way to have a successful program. It has been suggested that the size of the group remain between five and 15 participants for the most efficient discussions.^5^ It is suggested the arrangement of the members be in a circle to promote discourse.

#### 1.4.3 Definitions of success

The goal of any educational endeavor is success, but how this is defined may vary depending on the context. Alguire^15^ reports ways that journal clubs can be considered successful. The first is the longevity of the program, which is defined by having a continued active program for at least 2 yr, and the second is high resident participation (at least 50% participation). They found the greatest correlation to success with programs that required mandatory attendance, provided food for attendees, and was associated with smaller residency training programs.

When the medical residency program directors were asked what factors were involved in their level of satisfaction for the journal club, they mentioned the importance of regular attendance of the physicians in the department. Their attendance demonstrates the value of the meetings, not only the residents presenting but also the others in the department to promote the continued support of the educational experience. Interestingly, it was also mentioned that there was higher satisfaction from members if the meetings were held in a faculty member's home. Successful journal clubs have leadership for sustaining the program. Having a designated organizer of the meetings has been shown to increase satisfaction from the program directors and quality of the meetings. The clubs can be led by the director of the program or a faculty member for continuity throughout the lifetime of the program. Another option is for the sessions to be managed by a chief resident or small group of senior residents.

#### 1.4.4 Limitations

There are many challenges to establishing and maintaining a healthy journal club. Some of these challenges include a lack of time of both physicians and residents in the busy clinic. Because of this lack of time, even if a journal club is scheduled for an hour during the work week, the residents may not spend time preparing for the session. If there is insufficient preparation, the sessions are usually unstructured and are therefore not highly valued by the members decreasing educational potential for the participants.^18^ As mentioned earlier, it is challenging to establish continued interest and participation for the event if there are not clear goals for the meetings.^15^

#### 1.4.5 Discussion content

The content and the format of the journal club should reflect the goals that have been defined for the meetings. Alguire^15^ reports several different methods how the journal club can be established. The journal club could focus on one single paper with emphasis on meticulously presenting each of the sections of the article including the objectives, hypothesis, and conclusion. The resident can lead a discussion of the validity of the assumptions and conclusions mentioned in the specific paper and critique the methodology that was used.

Others recommend the use of several articles to give a wider overview of a topic or issue. Sadeghi and Kakhki^7^ suggest incorporating systematic reviews and meta‐analyses to provide a more objective presentation of the topic. Multiple articles could also be presented to compare and contrast the differences between the two in order to determine which author provides the best evidence for the case.

Whether one or several papers are discussed, Joorabchi^6^ suggests that the research discussed be experimental instead of just descriptive so open‐ended questions can be posed to the audience to provoke both small and large group discussions related to the given literature. Other formats include creating a controversy, reviewing classic articles, and using case‐studies to focus on problem‐based learning.^8^, ^19^

### 1.5 Scaffolding models

It has been noted in the literature that there are shortcomings to the traditional way journal club is conducted. The resident is often ill‐equipped to select an appropriate article for discussion because they have not been trained in how to conduct a search for a quality research article or assess the article critically.^18^ While some are trying to address this deficiency earlier at the undergraduate level, Pato et al.^17^ present a scaffolding model in which the residents can progress in their competency throughout their program by guidance by phases of critical thinking.

A resident should be able to evaluate the strengths and limitations of the article. One way of accomplishing this is to start by providing an outline for the residents to fill out with keys on how to identify important elements of the article. A paper may initially be selected with the help of a support committee, giving the resident several options for acceptable papers so the resident can focus their initial efforts on understanding and critiquing the content of the article.^4^ Another approach is to assign both a strong and weak article for the resident to evaluate.

One critical skill is being able to search and find an appropriate research article. This scientific literacy involves knowing how to navigate medical and scientific journals for relevant articles. Residents should be able to evaluate key parts of the paper like the title, author, references, and abstract when initially assessing a paper. Since the ability to read scientific journals is a life‐long learning experience, residents should learn how to efficiently sort the good articles from the bad early in their career. After finding quality articles, it is also a valuable skill to know how to organize and store the literature for future reference or for citing later when writing their own papers.^17^

The critical piece to the scaffolding model in gaining scientific literacy is the guidance from a mentor to help the resident gain progressive independence. While there are many role‐models in the clinic, it is often helpful to have a designated journal club leader or committee that can invest time and answer questions for the resident in a busy clinic.^18^ This role can be supplemented by advisors and other clinicians, but it is advantageous to have a dedicated point person for support so mentors can help clarify points of difficulty, help residents grow, and provide feedback throughout their residency.

### 1.6 Studies evaluating journal club effectiveness

There have been a few limited studies showing the effectiveness of journal clubs. These studies either rely on self‐reported perceived gains of the residents or by assessments without formal validation. Ebbert, Montori, and Schultz^20^ provide a systematic review of the studies which evaluates the effectiveness of journal clubs. Unfortunately, at the time of publication, there were only seven studies that met the search criteria for evaluation including a variety of study methodologies including one randomized controlled trial, cohort studies, a before and after study, and cross‐sectional studies. Almost all of the studies showed improvement in the resident outcome with the journal club intervention.

Most of the studies are based on self‐reports from residents. The one randomized controlled trial by Linzer^2^ assessed self‐reported resident skills. The results showed statistically better reported reading habits for those with the opportunity to participate in journal club. There was also measured improvement in clinical knowledge and use of medical literature in clinical practice, but there was no increase in critical appraisal skills. Sadeghi et al.^4^ measured residents' knowledge of evidence‐based medicine using self‐reported questionnaire. This study showed those residents involved in journal club displayed gains in knowledge of statistical significance, acquaintance with evidence rating, and familiarity with study design.

Another approach to evaluating journal club effectiveness is to provide an opportunity for residents to demonstrate gains instead of self‐reporting their perceived gains. Green^8^ had residents demonstrate critical appraisal skills by having them review a test journal article or answer a question based on a clinical situation. Another option is to assess resident behavior in the clinic to evaluate whether the skills are being applied to their daily practice or are simply compartmentalized to didactic knowledge. Unfortunately, it is not clear from these studies if the type or amount of discourse was a factor in resident gains.

### 1.7 Discourse within journal club

While it is stated that the goal of these meetings is to promote discussion among both residents and faculty, there is very little description or explanation of how this discourse takes place in this type of setting. Jamal makes the distinction that the session “is called ‘Club’ not a seminar”^3^ ^(p. 216)^ because it should involve the facilitator motivating and engaging participants. The opportunity for “open and free discussion is the heart of the journal club.”^3^ ^(p. 216)^ Journal club provides a unique opportunity for both residents and physicists to have social interaction where the faculty should be able to share their passion and excitement in introducing new members to the world of medical research and education.^5^, ^21^

Audience participation is key to a healthy journal club. The best education for the residents occurs when there is an exchange of ideas among the club members to stimulate learning and the synthesis of new information. One way suggested to promote this discourse is to arrange the participants in circle or round table so that participants can have eye contact and clearly communicate with others in the group.^19^ In this environment, all members should feel safe to communicate and exchange ideas.

Ways that are described in the literature to promote discourse in journal club include having meetings that are structured and well organized by the facilitator. Topics can be selected that are controversial in regards to claims or methodology presented in the article. Opposing viewpoints can also be introduced to encourage argument stimulating interest and discussion among the group members.^4^ By having diverse members present, there is greater opportunity for unique perspectives and therefore vigorous debate.^5^ Another strategy for promoting conversation is to ask questions to the participants to consider at the beginning of the session and discuss thoughts before presenting the findings of the author.

There are also negative factors that confine the contributions of members to the overall conversation. One limitation for discourse involves the skills and/or preparation of the facilitator of the session. Depending on the resident, the critical reading and presentation skills can affect the quality of the journal club session. Residents may not have sufficient background knowledge about the topic or could feel intimidated by contributing to the discussion for a variety of reasons.^17^ While the goal for the session is to be open and nonthreatening, residents can often feel anxiety when either presenting or contributing to journal club discussions.^18^

One of the most common challenges to discussion in journal club is the senior faculty members usurping the conversation.^3^ It is difficult for residents to have open discourse with the group when respected members of the department dominate the discussion. It should be a clearly defined expectation that participation is encouraged by everyone.^4^ Another strategy is for the faculty to have their own separate journal clubs, so that residents can have one that involves both faculty and residents dedicated to resident education.^17^

### 1.H Effect of technology and social media on journal clubs

The journal clubs discussed so far have taken place physically at an institution and are conducted face‐to‐face with the participants. However, with advances in technology, capabilities of social media, and necessity of thinking outside of traditions due to the COVID‐19 pandemic, the definition and venue for journal clubs is continuing to expand. Virtual meeting platforms, blogs, and social media give residents and doctors beyond the academic institutions a chance to participate in these discussions.^22^, ^23^ Professionals from all over the world have an opportunity to engage in discourse with colleagues about new publications. These conversations have the ability to present many perspectives and provide a forum for provocative questions to be asked by minds outside of one's own institution. Oftentimes, these spark interesting side conversations related to the main topic that can be further pursued outside of journal club.

Benefits for online journal clubs include increased access and democratic nature to information and discourse. With the accessibility of the internet, anyone can now access relevant and accurate information about medicine.^24^ Participants have a way of interacting and collaborating with experts from across the world. However, depending on the venue for the discussion, privacy of the conversation could be a concern to contributors. One way of hosting an online journal club is through Twitter where all users are aware that nothing written is private. Since all posts are public, participants understand that what they say can be accessed by anyone and should be mindful of their contributions.

As of 2015, there were 24 known medical journal clubs using Twitter. The online journal clubs are gaining in popularity with progressive increases in the number of followers and number of Tweets from users over time.^9^, ^25^, ^26^, ^27^, ^28^ This number has continued to grow during 2020 due to the pandemic. One example is a journal club for nephrology (#NephJC) that occurs twice a month, which is hosted through Twitter.^24^, ^29^ The group discusses interesting studies, reviews, and clinical practice guidelines in the medical specialty, and these participants meet at a designated time and the journal club last for about an hour.

Online journal clubs have been either arranged to occur at a certain time and date where people can participate together or have been conducted over a longer amount of time for asynchronous discussion. The latter has the advantage of allowing professionals from all over the world to contribute to the discussion at a convenient time. For example, the International Urology Journal Club (#uroJC) provides notification about the topic about a week before the scheduled meeting, and the asynchronous club meets for 48 consecutive hours.^24^ Since all of the discourse for the journal club is recorded through text, journal club organizers are able to easily maintain an archive of previous conversations.

The Medical Physics Leadership Academy launched their leadership journal club initiative in the fall of 2020.^30^ The venue is open to all American Association of Medical Physicists (AAPM) members via prior registration and has been hosted during a monthly Zoom meeting. Materials are preassigned prior to the session and breakout groups allow for discussion in a small group before the final large group discussion. A bulletin board system has also been developed for asynchronous conversation outside of the monthly meeting.

### 1.I Methods for increasing and varying discourse

While there have been some mention of strategies for ways that discourse can be incorporated in journal clubs from the literature, this section will provide some additional methods for these beginning or refining their own journal club. First the leader of the session should explain the rationale for the use of discourse in the journal club. The ground rules should be established for creating a safe environment for the exchange of ideas within the group. Both the facilitator and the members should demonstrate good listening skills and practice building responses on one another's comments.

Facilitators can increase opportunities for dialog by moving away from a traditional lecture and asking high‐quality open‐ended questions to the audience to promote discussion. Small groups can be tasked with specific questions for discussion or two people can work together as a think‐pair‐share team for stimulating discourse. The facilitator can also call on individuals for answers during the session. Members should be encouraged to provide reasoning and justification for their ideas which is consistent with evidence based medicine. The facilitator should always seek multiple answers and explanations from the group to promote involvement from each individual.

### 1.J Proposed model for journal club in medical physics residency

**Fig. 1:**
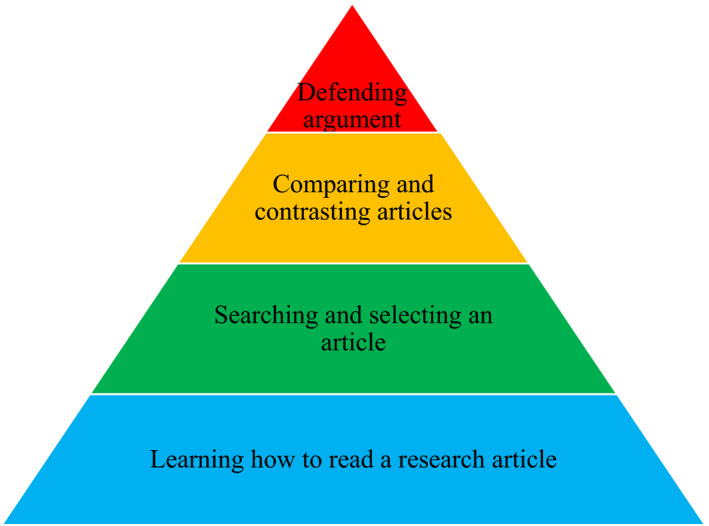
Four‐phase model for medical physics journal club.

We began a journal club for our medical physics residency in 2015 with the goal of promoting accountability for reading scholarly work in the field. After reflecting upon the literature, it has shown me that we should provide more than just a forum to present something that has been published in the form of an educational lecture. While our traditional model was a way of transmitting the knowledge to those within the department, we shifted the focus to mentoring residents and gradually building their skills through scaffolding. Teaching how to evaluate the quality of a research article and how to promote a meaningful discussion in a way that is more interactive will provide an environment that is more meaningful for the attendees. We have incorporated a four‐phase model to journal club in our residency program shown in Fig. 1.

1. **Review a research article using an outline for key aspects**. The resident will complete assigned readings reviewing journal club and review video on writing good scientific papers. The resident will identify a topic of interest and mentors will help identify a good paper on the topic. The resident will fill out a worksheet to help guide the understanding of the structure and purpose of the article. The resident will share what he has learned in a physics education session using active learning strategies.
2. **Learn how to search for and select a good journal article**. The resident will be introduced to scientific journals within medical physics and radiation oncology by reviewing videos on differences between journals in medical physics through the AAPM Virtual Library. They will be introduced to impact factor and the process of publication and review. The resident will also be exposed to citation management software and select one for download and use. The resident will select a topic of interest and propose three articles within a topic of interest. One final article will be selected after discussion with mentors for presentation to the group during the journal club meeting.
3. **Compare and contrast a strong and weak article on the same topic**. The resident will review videos from the AAPM Virtual Library discussing what journal article reviewers are looking for in a manuscript. The resident will select a topic of interest and review several articles within a topic of interest. The resident will rank the articles and select an example of a strong article and weak article and defend why they believe so. The resident will schedule a meeting with mentors to review and justify his choice. The two final articles will be compared and contrasted for presentation to the group using active learning strategies during journal club meeting.
4. **Research and defend one side of an argument in a point/counterpoint**. Resident will coordinate with another coresident to select a topic for a point/counterpoint discussion that will be held during the journal club. Any topic of interest may be selected, but topics are encouraged from the AAPM list of past articles, which can be found in either of the two volumes of “Controversies in Medical Physics: a Compendium of Point/Counterpoint Debates.”

This journal club model has been adopted by our residency program and educational materials have been developed such that it would be simple to expand the educational curriculum to other institutions. Appendices A and B provide supplementary reference material for beginning a new program or adapting a current program based on our current curriculum. After several years of implementation and refinement, we have received positive feedback from the residents for the guided approach in learning these valuable skills and feedback from the physicists in the increased quality of resident presentations using learner‐centered approaches. While this review presents a model for the educational program, work is ongoing work to help evaluate the efficacy of this educational approach. This four‐stage model can be expanded to many different audiences in the medical community as a format for not only physicists and physicians, but all health professions as a prototype for interacting with and presenting academic literature.

## 2 CONCLUSION

By the time a medical physicist completes his or her residency, he or she is well versed in the practice of their specialty. A universal skill desired and expected for all clinicians is the ability to retrieve, analyze, and present scientist knowledge from medical and research journals. While journal clubs may be different from institution to institution, there are core ideas that should define the quality educational experience the venue is intended to provide. This paper presented the idea that journal clubs are a valuable space for analyzing scientific literature, promoting analytical reading skills, and provides a setting for intellectual discourse. Several methods were described in how to define appropriate goals, select topics, and provide methods for conducting successful journal club experiences for resident education. Finally, discourse methods were highlighted for application in both a traditional and online setting.

There is currently very little literature in terms of how discourse is used within journal club settings. Future work can be expanded on how journal clubs are conducted with a focus on the conversation or lack thereof within residencies. By first knowing what kind of discourse is currently in the sessions, we can then expand to evaluating the effectiveness of types of discourse to improve the educational experience for medical residents. Finally, these findings can be generalized and tested within other settings like journal clubs at the undergraduate level.

## Figures and Tables

**Fig. 2:**
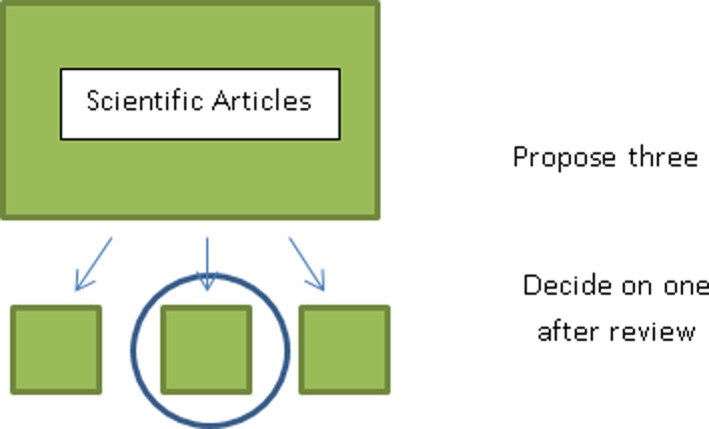
Example of resident proposal during journal club part II.

## APPENDIX A

### Example of journal club curriculum

#### Part I

1. Complete four readings about journal club. Examples include:
  - A Problem‐Based Journal Club. Bahman Joorabchi. *Journal of Medical Education*. 1984. Vol. 59. Pg. 755.
  - Journal Club for Faculty or Residents: A Model for Lifelong Learning and Maintenance of Certification. Michele T. Pato, Robert T. Cobb, Shari I. Lusskin, and Connie Schardt. *International Review of Psychiatry*. 2013;25(3), 276‐283.
  - A Journal Club Workshop that Teaches Undergraduates a Systematic Method for Reading, Interpreting, and Presenting Primary Literature. Katerine Roberson. *Journal of College Science Teaching*. 2012. 41(6), 25‐31.
  - A New Approach to Teaching and Learning in Journal Club. Khalid S. Khan and Harry Gee. 1999. *Medical Teacher*. 21(3), 289‐292.
2. Review AAPM Virtual Library video. Example includes:
  - Writing good scientific papers and responding to critiques — Mitchell Goodsitt (2016)
3. Setup Goggle Scholar to connect through institution's library system.
4. Identify a topic of interest and submit to residency preceptor to help you find articles you can choose from for reading based on your interests
5. Read the article and fill out template outline for reviewing articles. (See Appendix B for template.)
6. Schedule time to meet and review with preceptor
7. Prepare presentation to share information at Journal Club

#### Part II

1. Watch videos from the AAPM virtual library about journals in our field. Examples include:
  - ASTRO Based Journals — Eric Klein (2016)
  - Journal of Medical Physics and JACMP — Jeffrey Williamson (2016)
  - Journal of Applied Clinical Medical Physics: Mission and Submission — Susan Richardson (2015)
2. Practice searching for articles and reviewing the latest articles in Medical Physics, JACMP, PMB, etc.
3. Watch the following for an introduction to citation management software.
  Example: https://www.youtube.com/watch?v=sy9PVZAbSAQ

1. Watch the following for an introduction to citation management software:
  - Introduction
  - Review what is available through institution and other options.
2. Select one of the citation managers and download
  - Pick a few online tutorials to watch and get started using the reference manager
3. Identify a topic of interest and select three scholarly articles to submit to preceptor
  - We will review and select one shown in Fig. 2.
4. Schedule time to meet and review with preceptors
5. Prepare presentation to share information at a Journal Club

#### Part III

1. Watch videos from the AAPM virtual library about how to be a reviewer in our field. Examples include:
  - How to be a referee — John Boone (2012)
  - How to be a referee — Colin Orton (2012)
  - How to be a referee — George Starkschall (2012)
  - How to be a referee — Bruce Thomadsen (2012)
2. Identify a topic of interest and select two scholarly articles to submit to preceptors including your comparison and defense of why you believe one is a strong paper and the other is a weaker article.
3. Schedule time to meet and review with preceptors.
4. Prepare presentation to share information at a Journal Club.

#### Part IV

1. Resident will coordinate with another co‐resident or physicist to select a topic for a point/counterpoint discussion that will be held during the Journal Club. Any topic of interest may be selected, but topics are encouraged from the AAPM list of past articles, which can be found in either of the two volumes of “Controversies in Medical Physics: a Compendium of Point/Counterpoint Debates.”
2. Residents will research not only their side of the debate, but their opponent’s points as well. Examples for resources include:
  ○ Basic Argumentation:
    ▪ http://www.speaking.pitt.edu/student/argument/argumentbasics.html
    ▪ https://www.youtube.com/watch?v=l6_6i‐OJ_e4
  ○ Format: https://www.youtube.com/watch?v=Q5_nAtHh9Xk
3. Schedule time to meet and review with preceptros
4. Prepare presentation to share information at a Journal Club
  - The format will be based on that of Lincoln/Douglas Debate Format, but modified slightly:

#### Modified point/counterpoint structure

First affirmative constructive — 7 min

- A good introduction that attracts the audience’s attention and interest in the topic
- Clearly state the resolution
- Clearly state each of you contentions with reason and evidence
- Conclude effectively

Cross examination of the affirmative by the negative — 3 min

- Ask courteous questions

First negative constructive — 7 min

- A good introduction that attracts the audience’s attention and interest in the topic
- Clearly state the Negative’s position on the topic
- Clearly state the negative’s observations with reason and evidence
- Challenge and question affirmative’s contentions and evidence
- Conclude effectively

Cross examination of the negative by the affirmative — 3 min

- Ask courteous questions

Negative closing summary — 2 min

Affirmative closing summary — 2 min

## APPENDIX B

### Journal Club Review Form

Adapted from Jamal (2015), Pato et al. (2013), and Robertson (2012)

1. Observation that led to research
  - Describe 2–3 observations.
2. Question
  - State why the question is important (What did the authors hope to learn about the field?).
3. Hypothesis
  - What is the hypothesis of the paper?
  - Are the objectives clearly stated?
  - Explain why these hypotheses make sense based on current knowledge.
4. Methods
  - Describe general experimental design. (What was measured/compared?)
  - Describe the methods and controls in your own words.
  - Choose 2–3 key figures that directly address the hypotheses.
  - Describe the study design. (Examples: correlational, case report, case series, cross‐section, cohort, case control, experimental, meta‐analysis, RCT, Review, other)
  - Time frame: (examples: prospective, retrospective, n/a)
  - Randomized: (examples: random, nonrandom, n/a)
  - Blinded: (examples: unblinded, single blinded, double blinded, n/a)
  - Enrollment: (examples: convenience, consecutive, other, n/a)
  - Explain why the choice of controls was (or wasn't) appropriate.
  - Describe statistical tests used in study:
5. Results
  - Explain figures clearly. Restate in your words what is being compared to what for each one.
  - Describe trends: (example: What is increased over what?)
  - Identify the controls and how they validate the trends.
  - Look for statistical analyses (figure legend or results) that validate the data.
6. Discussion
  - Was there bias in the study? If so, where?
  - Who or what can the results be generalized to?
7. Conclusion (based on the data, not on the discussion)
  - Does the data support the hypotheses?
  - Are there other possible explanations for the data?
  - Is the data convincing?
  - How could the experiment be improved?
  - Why is the data interesting? How does it contribute to our understanding within the field?
8. Will you change your clinical practice based on this study? If so, how? If not, why not?
